# Supplementary material for: Are carbon emissions trading and green financial instruments synergistic? -Comprehensive quantitative research based on content analysis
Source: PLoS One. 2024 Mar 7;19(3):e0298601. doi: 10.1371/journal.pone.0298601 (PMC10919601; doi:10.1371/journal.pone.0298601)
Supplement: S1 Data — (ZIP) [file pone.0298601.s001.zip › Data/地级市Z_F工作报告.zip!/╣π╢1⁄2/│▒╓▌╩╨/└┤╫╘╣1⁄2╓┌║┼í╛┬φ┐╦╩2╛▌═°í┐_2015.docx]

各位代表：

现在，我代表市人民政府向大会作工作报告，请予审议，并请市政协委员和其他列席人员提出意见。

2014年工作回顾

过去一年，我们在省委省政府和市委的正确领导下，在市人大和市政协的监督支持下，紧紧依靠全市人民，围绕加快振兴发展的主题，沉着应对复杂严峻的经济形势，扭住交通扩网提速、园区扩能增效、城市扩容提质“三大抓手”，着眼创建粤东生态环境和社会治安最佳城市，扎实做好稳增长、调结构、促改革、惠民生等各项工作，全市经济社会实现持续稳定发展。

综合实力稳步提升。全市经济稳中向好，主要经济指标增幅超过全省平均水平。地区生产总值完成850.2亿元，比上年增长8.2%。地方公共财政预算收入41.3亿元，增长11.3%。税收总收入91.4亿元，增长3.7%。发展后劲持续增强。固定资产投资完成313亿元、增长30.1%，其中基础设施投资89.8亿元、增长59.6%，工业投资129.7亿元、增长4.8%。年度72个市重点项目完成投资106.2亿元，占年度计划的95.2%。市场消费保持畅旺。社会消费品零售总额395.8亿元，增长11.8%。外贸出口28.5亿美元，增长2.5%。人民生活不断提升。城乡居民平均收入预测增长10.5%，居民消费价格指数上涨1.5%。

城乡建设扎实推进。城市扩容提质有序开展。各类规划编制力度加大，中心城区控规实现全覆盖，潮州新区4个专项规划编制完成。韩东新城起步区启动实施，27个新区重点项目已开工11个、年度完成投资21.8亿元，韩江东岸综合开发项目进入实质性建设阶段，潮州恒大城、东方国际茶都、市中心医院新院等项目加紧推进，新区建设初见成效。古城区部分街巷改造全面完成，新天地项目加快实施，城市燃气“一张网”建设着手开展，绿化美化水平不断提升，我市顺利通过国家园林城市省级复查。交通体系逐步优化。厦深高铁潮汕站配套建设不断完善，功能定位得到提升，区域中心枢纽站地位进一步确立。韩江大桥维修工程、潮汕火车站进站公路、饶平环城北路等项目基本完成，潮惠高速、护堤公路、潮州大桥、韩江东西溪大桥、潮州港扩建货运码头建设扎实推进，宁莞高速公路潮州段开工建设，梅汕铁路、大潮高速等项目前期工作有效开展。园区支撑能力增强。中山（潮州）产业园获得省专项扶持资金2.5亿元，开发建设、招商引资、项目落地进展顺利，产业总量进一步做大。闽粤经济合作区发展规划通过专家评审并上报省政府，已争取写入省政府工作报告，并努力争取成为泛珠三角国家战略的重要合作平台。新农村建设加快推进。省级新农村示范片落地建设，三大涝区整治取得新进展，小型病险水库除险加固4座、堤围加固20.4公里，新改建县乡公路43.6公里、通村公路215公里。生态环境建设加强。新建森林碳汇工程7.68万亩、生态景观林带55公里，新增森林公园18个、绿化美化乡村182个，粤东柘林湾海洋生态修复工程全面实施。违法用地和违法建设有效遏制，资源节约集约利用水平提升。水源和大气污染防治积极开展，桥东污水处理厂配套管网改造工程完工，淘汰“黄标车”1.04万辆，单位生产总值能耗完成年度控制目标。

产业结构调整优化。发展质效进一步提升，三次产业结构优化为6.9：56.4：36.7。工业升级步伐加快。潮州—中山产业创新创意园投入运营，市、区、镇、企四级创新联盟成效明显。新增省级企业技术中心5家、工程研发中心2家，新参与各类标准制修订24项，专利申请量和授权量均居全省第10位，新增省级以上名牌名标11项，新认定高新技术企业15家，三环集团成功上市，粤金曼破产重组进展顺利，陶瓷灯饰业态培育壮大，我市被认定为“陶瓷国家新型工业化产业示范基地”和“中国工艺鞋出口基地”，饶平钱东镇被授予“中国盐焗鸡之乡”。全市规模以上工业增加值完成332.5亿元，增长11.1%；产值超亿元企业达到350家，同比增加56家。农业生产全面发展。粮食生产增产增收，茶叶、蔬菜、花卉、水产等产量持续增长，佛手果被评为广东名果。高标准农田建设积极实施，三百门国家一级渔港建设顺利完成。农业产业化水平提高，“菜篮子”工程基地和农业龙头企业引领作用增强，农民专业合作社增至713家。服务业进一步提升。旅游经济发展壮大，新增国家4A级景区3个，全年接待海内外游客758.1万人次，实现旅游收入115.9亿元，分别增长28.4%和29%。商贸物流业和电子商务获得新发展，广东海润冷链物流一期工程全面启动，三百门码头进口肉类指定口岸通过验收，枫溪陶瓷工贸进出口公司被认定为省外贸综合服务培育企业。

改革开放不断深化。市场化导向的经济领域重点改革全面推进，发展活力进一步迸发。商事制度改革成效突出。全市新登记市场主体12386户、个转企424家，其中新登记私营企业1821家。金融创新扎实开展。粤东西北振兴股权发展基金对接落地，新增村镇银行2家、小额贷款公司3家，饶平县被省列入农村普惠金融试点县。全市金融机构本外币存款余额首次突破1000亿元，存贷比为35.6%。农村综合改革逐步深化。农村土地承包经营权确权试点工作启动实施，涉农维稳重大事件的预防、排查和化解工作扎实开展。对外开放务实推进。与中山市对口合作成效扩大，对外交流合作不断深化。招商引资力度加大，全年实际利用外资1.09亿美元、增长8.1%。

社会事业全面进步。民生福祉得到改善。十件民生实事基本完成年初预期目标，财政投入达到20.95亿元。底线民生保障政策全面落实，社保、低保、五保待遇和残疾人补贴提高，住房保障力度加大，市残疾人康复中心竣工，新一轮扶贫开发取得阶段性成效。就业社保工作得到加强。新增城镇就业25013人，失业人员再就业4027人，组织劳动力培训、转移就业12689人和25311人，城镇登记失业率2.47%。社会保险服务模式创新、覆盖面扩大、待遇水平提升，城乡居民基本养老保险、医疗保险参保率分别达到99%和98%。社会事业全面发展。教育创强工作加快推进，公办义务教育标准化学校覆盖率达到100%。文化惠民工程积极实施，道德讲堂、文明社区先行点创建工作扎实开展，新增国家级非遗项目3项，《潮州市志（1992－2005）》出版发行，我市输送的运动员在亚运会、全运会夺取多枚金牌。人口计生服务管理不断加强，“单独两孩”政策得到落实，人口自然增长率为5.5‰。医药卫生体制改革继续推进，基本公共卫生服务均等化水平提高，登革热、禽流感等传染病有效防控，省级卫生镇创建实现零的突破。社会服务管理深化。食品药品监管体制改革全面推进，一村（社区）一法律顾问试点工作有效开展，村居“两委”顺利换届。“社会矛盾化解年”活动扎实开展，消防安全管理加强，安全生产和应急管理水平稳步提升，“平安潮州”创建成效明显，信访突出问题化解率、路面执勤警力到位率、路面管事率和处警及时率均达到100%，群众安全感和对政法工作满意度居全省前列。与此同时，人事、统计、法制、外事侨务、贸促、物价、粮食、打私、打假、人防、气象、防震、档案、对台、拥军优抚、民兵预备役、老龄、民族宗教、妇女儿童等工作也取得新成绩。

法治政府建设加强。依法行政深入推进。接受人大、政协监督意识增强，人大代表建议42件、政协提案82件均按期办结，满意率和基本满意率达到99.2%。政府职能转变步伐加快。市县区政府机构改革全面完成，潮州新区管委会正式运作。行政审批标准化建设扎实开展，新调整行政审批事项41项。网上办事大厅拓展完善，网上办事办结率达到96.7%。廉政建设不断强化。市级考核项目大幅减少，政务公开、行政监察、审计监督、网络问政、纠风工作力度加大。党的群众路线教育实践活动深入开展，会议费和“三公”经费削减25.3%。

各位代表，上述成绩来之不易。这是省委省政府和市委坚强领导的结果，是全市人民艰苦奋斗的结果，是市人大、市政协监督支持的结果，也是驻潮部队、武警官兵、上级驻潮各单位和海内外各界人士鼎力支持的结果。在此，我代表市人民政府表示衷心感谢并致以崇高敬意！

在看到成绩的同时，我们也清醒地认识到，我市发展中还存在不少问题，主要有：经济总量偏小，产业结构不优，产业龙头企业偏少，经济下行压力较大；城市综合管理水平不高，公共设施配套不足，城市面貌亟待改观；土地、资金、人才、资源环境等要素制约突出，大气环境质量有待改善，节能减排任务十分艰巨；政府服务效能还需提升，机关作风建设仍需加强。对此，我们一定高度重视，下大力气采取措施加以解决。

2015年工作安排

今年是全面完成“十二五”规划的收官之年，也是潮州全面深化改革、推进依法治市、加快提速发展的重要一年。当前，世界经济仍处在深度调整期，总体复苏态势疲弱；我国进入经济发展新常态，将从高速增长转向中高速增长。与此同时，我市经过20多年的发展，已积累了大量加快发展的经验和要素。对此，我们必须适应新常态，探索新思路，激发新活力，敢于新作为，把潮州加快提速发展这篇文章做好。今年政府工作总的要求是：全面贯彻党的十八大和十八届三中、四中全会，以及省委十一届四次全会和市委十三届五次全会精神，继续抓好省振兴粤东西北战略机遇，围绕建设“一中心三片区”战略定位，牢固把握加快提速发展的思想导向，认真编制全域发展规划，坚持“三大抓手”引领，全面推进改革创新，加快产业转型升级，发展壮大实体经济，加强生态文明建设，提升公共服务水平，维护社会和谐稳定。

综合各方面因素，今年经济社会发展主要预期目标是：生产总值增长11%、力争达到11.5%，地方公共财政预算收入增长15%，固定资产投资增长25%，外贸进出口增长4%，社会消费品零售总额增长13%，居民消费价格指数涨幅控制在3%以内，城镇登记失业率控制在3%以下，居民收入增长与经济增长基本同步。

上述目标是根据中央和省的总体要求，综合考虑质量效益导向、远近目标衔接、振兴发展需要等多方面因素，经过反复论证和审慎决策确定的，有基础、有可能；但在宏观调控、市场变化、资源约束等倒逼因素较强的情况下，也有难度、有压力。实现这些目标，必须根据需要与可能，分清轻重缓急，集中财力、物力、人力解决事关发展大局的问题。重点把握三条原则：一是产业发展突出导向性。根据我市现实情况、产业基础及未来趋势，通过支持民营企业再创业，推动民营经济再出发，优化提升现有特色产业，培植发展新的产业集群，做强产业存量，做大产业增量。在整体布局上，加强规划引领，高水平编制各产业发展专项规划，集中建设一批产业集聚区。在发展模式上，坚持创新驱动战略，突出抓好产业园区、主导产业、龙头企业三大载体，力促产业向规模化、高端化、集群化发展。在推进路径上，坚持非均衡配置资源，集中有限的要素集约投放，推动重点产业、重点企业做强做大。二是基础建设把握关键性。我市财力有限，能调动的资源不多，基础建设不可能全面开花，必须紧紧依托“三大抓手”，分享政策红利，突破一些瓶颈和制约。交通建设方面，围绕建设立体化、网络化交通体系，通过交通大会战，着重在高速铁路、高速公路、港口运输、国省道改造上发力。园区建设方面，围绕做大园区经济总量，着重完善园区设施、提升承载能力，形成更大的投资规模。城市建设方面，围绕城市东扩目标，着重推进潮州新区起步区建设，尽快启动沿潮州东大道的征地拆迁，争取有一批重大项目布点落地。三是民生工作坚持原则性。改善民生没有终点，只有新起点。必须按照“守住底线、突出重点、兼顾全面”的思路，坚持量力而行、多元化投入，把关系群众切身利益的热点难点问题作为工作重点，集中力量突破薄弱环节，实现普惠政策全覆盖、困难特例兜住底，真正把民生实事办到群众心坎上。

围绕今年总目标，重点做好以下六方面工作：

一、全力发展壮大实体经济

实业是立市之基、强市之本，必须坚持增量与存量并重，增强工业支撑作用，加快发展实体经济，努力把经济堆头做大。

做强存量。坚持创新驱动发展，实施品牌和标准化战略，开展质量提升行动，编制技术改造导向计划，支持企业技术改造、加强管理和科技创新，推动企业从生产制造环节向价值链高端延伸。完善创新服务体系，推进国家知识产权试点城市工作，加强专业镇、四级创新联盟、质检中心等平台建设，培育科技企业孵化器，深化产学研合作，组织实施“陶瓷生产智能机器人”项目，支持企业建设“人才池”，促进产业转型升级，实现产业“凤凰涅槃”。出台鼓励扶持措施，集中资源要素，支持民营企业诚信规范发展、加快做强做大。

做大增量。构建多元化融资体系，活跃民间投资，培育市场主体，推动民营企业再创业，支持企业加大固定资产投资，鼓励帮助困难企业走出困境，促进中小微企业协同发展。深入实施大企业培育工程，推进个转企、小升规、规改股、股上市，引导上市企业做大规模，力争产值超亿元企业继续大幅增加、新上市企业2家以上。打好招商引资攻坚战，注重开展产业链招商，积极吸引和承接产业转移，延伸产业链条，培育发展陶瓷新材料、燃气生产、机械设备等配套产业，大力发展电子机电、生物制药、节能环保、新光源等新兴产业，打造2－3个新产业集群。

做优临港。突出潮州港的带动作用，完善港口港区配套设施，加快土地征收利用，推进三百门新港区综合开发、广东海润冷链物流中心等项目建设，抓好潮州港口岸查验服务中心项目前期工作，打造良好投资发展载体。加快引进一批高端化、高质化、高新化临港工业项目，推动粤东名贵木材集散基地、潮州港液化天然气粤东储备站、怡华油品仓储、粤东天然气配送中心等项目落地建设，争取大唐电厂3期工程开展前期工作，建设潮州港物流、产业集聚基地，努力做大港口经济。

做实三产。提升壮大旅游产业，力争接待海内外游客突破900万人次。启动全市旅游发展规划修编，出台加快旅游业发展的意见，积极创建省旅游综合改革示范市并申报国家旅游综合改革试点市。坚持历史文化名城保护规划引领，加强潮州古城文化旅游特色区规划建设，创新运营管理体制，突出打造2－3条特色街区，启动创建国家5A级景区。完善提升凤凰山生态旅游区、绿岛山庄、紫莲山庄等景区景点，发展乡村旅游和农家乐，提高旅游经济效益。办好旅游宣传推介主题活动，扩大城市整体形象营销和对外合作。加强高星级酒店等旅游配套设施建设，促进旅游和其他行业融合发展。积极培育新型文化业态，发展壮大木雕、潮绣、手拉壶等工艺美术产业规模。大力发展现代服务业，扶持建立原材料供应市场，加快厦深高铁商贸物流中心等项目建设，新建和提升一批农贸市场，完善城乡市场体系。创新连锁经营、物流配送等服务方式，加强电子商务服务体系及发展平台规划建设，培育形成一批电商产业园区。

二、全力推动基础设施建设

集中各方力量，优化资金投向，突出“三大抓手”，完善协调服务机制，破解项目建设难题，实施年度74个市重点项目，力争完成投资162亿元，引领扩大投资规模，夯实经济发展基础。

大力实施交通扩网提速。推进22个交通重点项目建设，完成年度投资40亿元以上。扩建厦深高铁饶平站，完善潮汕站功能，力争动车运用所落地。推进汕梅高铁、厦深联络线建设，做好疏港铁路前期工作。协调推进宁莞高速、潮惠高速潮州段建设，争取动工建设大潮高速公路。加快国道G324线、省道S233线等一批国省道改造工程，拓宽与周边城市的对接通道。动工疏浚潮州港公用航道，加快潮州港扩建货运码头工程建设。

大力实施园区扩能增效。加快构建省际合作战略平台，争取省政府尽快批准闽粤经济合作区规划建设，并纳入国家“十三五”发展规划，同时谋划和启动先行合作项目。推进中山（潮州）产业园扩容建设，抓紧各分园开发完善，有序开展土地征收，做好径南分园远期3.2万亩总体规划编制，拓展园区发展空间。加强园区招商服务，力促中山火炬（潮州）产业园、中加（国际）高新科技环保装备制造产业园等重大项目落户，推动入园项目落地建设。以各类园区为依托，连结周边专业镇，打造一批配套程度高、规模优势突出、核心竞争力强的特色产业集聚区。

大力实施城市扩容提质。启动城市总体规划修编，推进各类规划衔接整合，严格执行各项规划。完善潮州新区管理机构。加快韩东新城起步区建设，用好股权基金等资金，启动征地拆迁工作，动工建设潮州东大道，继续推进韩江东岸综合开发、潮州恒大城、潮州新天地、市中心医院新院等项目，争取完成意东堤、东厢堤防渗处理工程。加大招商引资力度，引入有实力的民间资金参与韩东新城建设，鼓励有条件的企业到新城建设总部大楼，促进更多产业项目落户。完善老城区功能配套，继续推进市区破损道路改造，加快潮州大桥、韩江东西溪大桥建设，动工建设外环西路潮安段。积极整治城市内涝多发区，推进“三旧”改造项目，尽快开展城市燃气“一张网”建设。实行城市网格化管理，加强交通拥堵整治，开展户外广告、占道经营等专项治理，巩固提升环卫市场化运营水平。

大力实施环境扩绿创优。推进新一轮绿化潮州大行动，划定林业生态红线，建好森林碳汇、生态景观林带、森林进城围城和乡村绿化美化四大工程。扎实抓好城乡环境卫生综合治理，全面改变“脏乱差”状况。巩固提升韩江综合整治成果，继续开展黄冈河、枫江治理。打好减排攻坚战，严格污染源头控制，推进5个污水处理厂建设。抓好大气污染防治，加强工业废弃物无害化处置，实现经济与环境和谐发展。加大耕地保护力度，推进高标准基本农田建设。强化土地利用计划管理，探索建立存量土地盘活、土地产出效益与新增建设用地计划指标分配挂钩制度，促进节约集约用地。加强河砂开采、销售环节监管，规范矿产资源管理秩序，科学利用海域资源。抓好电机能效提升，加快循环经济发展，促进节能降耗。

三、全力做好农业农村工作

坚持把解决好“三农”问题作为工作的重中之重，夯实农业稳定发展基础，稳住农村持续向好局势，不断增加农民收入。

推进农业现代化。加快转变农业发展方式，创新农业生产经营体制，推进农业产业化、规模化、标准化、品牌化发展，做大单丛茶、潮州柑、铁皮石斛、狮头鹅等优势特色农业。开展养殖用海市场化试点，大力发展深水网箱养殖等设施渔业。落实强农惠农政策，加强农业技术创新和推广，保障农产品质量安全，稳定粮食和主要农产品产量，促进农业增产增收。

推进新农村建设。统筹做好村庄整治、农房改造、农田水利、村村通自来水、历史文化村落保护利用等工作，抓紧实施引韩济饶工程和一批涝区整治，加快“一县一场”生活垃圾处理设施建设，设置固定的农村保洁人员，推进农村生活垃圾减量和无害化处理，积极创建省级新农村示范片、幸福村居、名镇名村示范村，为农民创造良好生产生活条件。

推进新型城镇化。健全城乡协调发展的体制机制，努力构筑市区一体、市县联动、资源共享的新型城乡关系。深化城乡空间形态研究，开展市域生态控制线规划编制。创新县城发展机制，加快中心镇、中心村建设。探索户籍制度改革，有序推进农业转移人口市民化。落实促进农民增收长效机制，加强农村实用人才培训。深化农村各项改革，做好农村土地承包经营权确权登记颁证工作，加强农村“三资”管理服务平台建设。

四、全力增创新的竞争优势

坚定不移推进改革创新，加快对内对外开放，统筹各类资源要素，补足发展短板，激活内生动力。

抓好潮州发展战略实施。认真编制全域发展规划和“十三五”发展规划，明晰“一中心三片区”战略定位，塑造“海丝文化重镇、潮人精神家园”城市形象，科学引领潮州发展。重点是将涵盖湘桥区、枫溪区的城市中心区，以韩东新城开发建设为引擎，打造成为全市首善之区；将涵盖潮安南部各平原镇的潮安片区，以高铁新城开发建设为引擎，打造成为粤东县域科学发展排头兵；将涵盖饶平中部和沿海的饶平片区，以闽粤经济合作区开发建设为引擎，打造成为闽粤台融合发展桥头堡；将涵盖潮安和饶平北部山区12个镇的北部片区，以绿色生态示范区开发建设为引擎，打造成为绿色经济发展示范区。

抓好经济领域改革发展。研究制订民营经济促进措施，鼓励民间资本以多种方式进入基础产业、社会事业以及特许经营领域，健全扶持本地民营企业发展和在外潮籍民企回归创业的工作机制，促进民营经济发展壮大。深化国有企业改革，完善国有资产管理体制，积极发展混合所有制经济。促进财税体制改革，改进预算管理制度，规范税收征收管理，统筹使用财政资金，设立市“偿债基金”。加快金融改革创新，推动农信社改制工作，积极吸引民营银行、产业基金等新兴金融机构落户，做好非国有建设用地等资源使用权抵押贷款文章。鼓励银行加大信贷投放，争取存贷比有所提高。加强政府融资平台管理，着力防范债务风险。做大土地储备规模，提高土地保障能力，发挥融资功能。用好用活特许经营等政府性资源，实现价值更大化。继续推进社会信用体系和市场监管体系建设，开展不动产统一登记发证工作，规范经济管理秩序。

抓好行政管理体制改革。理顺权责利关系，进一步简政放权，探索形成利益分配机制，调动各方面的积极性，形成强大的发展合力。完成工商、质监部门管理体制调整工作。全面清理行政职权和非行政许可审批事项，编制政府权责清单，推行负面清单管理，促进行政权力规范高效运行。规范行政审批项目办理标准和模式，所有审批项目统一纳入网上审批和电子监察系统。大力推行一站式服务制、一口收费制和检查报备制。加快延伸网上办事大厅，全面铺开镇、村办事站点建设。继续完善为民服务全程代理制。加强村、社区基层建设，创新基层社会管理模式。推进公共服务社会化、市场化，实行政府向社会购买公共服务。探索开展行政机关标准化管理。完成全市党政机关公务用车制度改革。

抓好对外合作空间拓展。加快外贸发展方式转变，建好陶瓷国家质量安全示范区和中国工艺鞋出口基地等公共平台，培育自主出口品牌和优势企业。健全外贸服务和风险预警机制，加快保税仓建设运营，组织企业参加广交会、中博会等经贸活动，支持企业加强与“海上丝绸之路”沿线国家和地区的贸易合作，巩固发展出口市场。深挖进口潜力，扩大先进设备等生产性资源和消费类商品进口，申报建立进口商品交易中心。加大招商引资力度，办好粤东侨博会，筹划设立驻外商务处，争取在香港、深圳、厦漳台、东南亚和欧美等地分别举办招商推介活动，推进区域经济合作，提升利用外资的质量和水平。深化与中山市的对口帮扶关系，促成更多合作成果。促进汕潮揭同城化发展，加强与海西经济区的交流合作。推进“广货网上行”，支持企业利用电子商务拓宽营销渠道，扩大与国内各类经济体的联合与合作。

五、全力提升公共服务水平

坚持兜底线、保重点，不断提升公共服务水平，增强群众幸福感。

保障底线民生。进一步加大财政投入，提高底线民生保障水平。2015年1月起将城镇和农村低保补差水平分别提高到374元/月和172元/月；五保对象供养标准提高到所在县区上年度农村居民人均纯收入的60%以上，孤儿集中供养水平提高到1240元/月，分散供养水平提高到760元/月；残疾人生活津贴提高到1200元/年，重残护理补贴提高到1800元/年；城乡医疗救助人均补助标准提高到1556元/年。

做好就业保障。落实各项收入增长措施，稳步增加城乡居民收入。深入实施更加积极的就业政策，健全公共就业服务平台，推动劳动力技能晋升培训。鼓励创业带动就业，完善创业小额贴息担保贷款工作机制，建设大学生创业孵化基地。以全员足额参保为目标，推进社保扩面征缴，健全社会保障机制。

统筹社会事业。继续推进教育创强工作，促进各类教育优质均衡发展。加强博士后科研工作站建设服务，培养引进高层次人才。推进全国文明城市创建。大力实施文化惠民工程，注重培养城市精神，筹划市科学馆、市体育中心建设，举办首届文化艺术节。重视文物保护和利用。继续深化医药卫生体制改革，健全公共卫生服务体系。实施优生健康惠民工程，促进人口长期均衡发展。抓好基层基础工作，强化食品药品网格化监管，依法打击各类违法生产经营行为，保障群众饮食用药和消费安全。认真开展1%人口抽样调查，继续做好民族宗教、外事侨务、广播电视、新闻出版、拥军优抚、国防动员、预备役、对台、人防、气象、防震、物价、档案、地方志、老龄、妇女儿童、残疾人、红十字等工作，促进各项事业全面发展。

建设平安潮州。不断完善立体化社会治安防控体系，夯实基层防控基础，努力构建“大防控”格局。集中优势警力实施严打整治，促进社会治安持续稳定，不断提高人民群众安全感和满意度。加强各类矛盾纠纷排查化解，扎实抓好信访维稳工作。牢固树立安全发展理念，强化交通、消防等安全管理，深入开展隐患排查治理，坚决遏制重特大生产安全事故发生。

办好民生实事。坚持民生优先、急需先行，集中力量办好十件民生实事。一是办好社会保障实事。2015年7月起将城乡居民基础养老金标准从每人每月80元提高至100元；2015年1月起将城乡居民医疗保险补助标准从年人均320元提高到380元；城乡居民医疗保险报销比例A档提高5个百分点、B档提高3个百分点。二是办好就业实事。新增城镇就业1.5万人、失业人员再就业4000人，促进创业1200人，城镇登记失业率控制在3%以内。组织开展劳动力技能晋升培训8000人。三是办好教育实事。将城乡免费义务教育小学、初中公用经费补助标准每年每生分别提高到1150元和1950元。将中等职业学校和普通高中国家助学金标准每年每生提高到2000元，对市县属中等职业学校免学费补助标准每年每生提高到3000元。四是办好医疗卫生实事。开展儿童口腔疾病综合干预项目，完成5000个小学生牙齿窝沟封闭。提升基层医疗机构综合服务能力，力争饶平县、潮安区各有1家县级综合医院达到二级甲等标准，有10所乡镇卫生院达到群众满意的乡镇卫生院建设标准。五是办好文化体育实事。加强公共电子阅览室公益性互联网服务，全面实现一村一月放映一场电影。加强基层健身场所建设，全市各镇均建有占地3000平方米以上的健身广场，以及使用面积累计600平方米以上的室内活动场所。六是办好住房保障实事。完成2909户农村低收入住房困难户住房改造任务。建设城镇保障房400套，发放租赁补贴800户。七是办好生态环境实事。新建森林碳汇工程7.68万亩、森林公园9个、湿地公园1个、森林家园34个，新增绿化美化乡村183个。八是办好交通实事。建立完善“智能交通指挥中心”，扎实推进彩虹东路、城新西路等道路贯通工程，缓解城区交通压力。新改建通村公路210公里，改善农村出行条件。九是办好社会援助实事。全面完成新一轮扶贫开发工作。创建残疾人维权工作达标市，实施残疾人“阳光家园计划”。加大对低收入群体、特殊困难群体的社会救助力度，推进医疗救助“一站式”即时结算服务。十是办好法律服务实事。全面开展一村（社区）一法律顾问工作，对全市1013个村（社区）按每村（社区）每年1万元的标准予以补助，并试点建设法律服务实体平台，为符合条件的困难群众提供法律援助。

六、全力建设人民满意政府

打铁还须自身硬，加强政府自身建设至关重要。我们将以改革创新为统领，改进工作作风，提升工作效率，全力建设人民满意的服务型政府。

坚持依法行政。深入贯彻十八届四中全会精神，强化法治理念、法治思维和规则意识，严格按照法定权限和程序行使权力。自觉接受人大、政协和社会监督，认真执行人大及其常委会各项决议决定，及时办理人大代表建议和政协提案。规范决策程序，完善议事规则，聘请政府顾问，借助外脑智慧，科学依法决策。推进行政执法规范化建设，落实重大行政处罚备案制。强化政府层级监督，坚决纠正违法或不当行政行为，促进社会公平正义。

持续改进作风。扩大党的群众路线教育实践活动成果，坚决遏制“四风”，着力解决“庸懒散”等突出问题。健全领导干部基层调研机制，完善领导干部直接联系和服务群众制度，深入听民意、问民需、解民忧。切实改进文风会风，大力精简会议文件。规范公务接待，加强审计监督，厉行勤俭节约，反对铺张浪费。

狠抓工作落实。坚持求真务实，细化目标任务，明确责任主体，制订进度要求，加强督促检查，确保工作到位。建立政府绩效监督和评估机制，形成正确的政绩导向，在干部中形成敢想、敢闯、敢干、敢负责的干事创业氛围，推动各项工作落到实处。

加强廉政建设。坚持用制度管权管事管人，进一步落实党风廉政建设责任制，健全惩治和预防腐败体系，完善政务和各领域办事公开制度，严肃查处各类违法违纪案件，让每一名政府工作人员切实做到守规矩、重纪律、知所惧，以廉洁勤政的作风，不断开创潮州各项事业发展新局面。

各位代表，深化改革迸发新的活力，法治思想凝聚新的共识，振兴发展迎来新的机遇。让我们在省委省政府和市委的正确领导下，解放思想、效率优先，凝心聚力、求真务实，以目标导向、用结果说话，共同为加快潮州振兴发展而不懈努力！
